# Evaluation of a 3D Printed Silicone Oral Cavity Cancer Model for Surgical Simulations

**DOI:** 10.3390/jpm14050450

**Published:** 2024-04-25

**Authors:** Donovan Eu, Michael J. Daly, Stefano Taboni, Axel Sahovaler, Ashley N. Gilbank, Jonathan C. Irish

**Affiliations:** 1 Guided Therapeutic (GTx) Program, Princess Margaret Cancer Centre, University Health Network, Toronto, ON M5G 2C4, Canada; michael.daly@uhn.ca (M.J.D.); axel.sahovaler@nhs.net (A.S.); ashley.gilbank@rmp.uhn.ca (A.N.G.); 2 Department of Otolaryngology-Head and Neck Surgery-Surgical Oncology, Princess Margaret Cancer Centre, University Health Network, University of Toronto, Toronto, ON M5S 1A1, Canada; 3 Department of Otolaryngology-Head and Neck Surgery, National University Health Systems, Singapore 119228, Singapore; 4 Section of Otorhinolaryngology-Head and Neck Surgery, Department of Neuroscience, “Azienda Ospedale Università di Padova” University of Padua, 35122 Padua, Italy; 5 Department of Oral and Maxillofacial Surgery, University College London Hospitals, London NW1 2BU, UK

**Keywords:** surgical simulation, oral cavity cancer, head and neck oncology

## Abstract

Adequate surgical margins are essential in oral cancer treatment, this is, however, difficult to appreciate during training. With advances in training aids, we propose a silicone-based surgical simulator to improve training proficiency for the ablation of oral cavity cancers. A silicone-based tongue cancer model constructed via a 3D mold was compared to a porcine tongue model used as a training model. Participants of varying surgical experience were then asked to resect the tumors with clear margins, and thereafter asked to fill out a questionnaire to evaluate the face and content validity of the models as a training tool. Eleven participants from the Otolaryngology-Head and Neck Surgery unit were included in this pilot study. In comparison to the porcine model, the silicone model attained a higher face (4 vs. 3.6) and content validity (4.4 vs. 4.1). Tumor consistency was far superior in the silicone model compared to the porcine model (4.1 vs. 2.8, *p* = 0.0042). Fellows and staff demonstrated a better margin clearance compared to residents (median 3.5 mm vs. 1.0 mm), and unlike the resident group, there was no incidence of positive margins. The surgical simulation was overall useful for trainees to appreciate the nature of margin clearance in oral cavity cancer ablation.

## 1. Introduction

Oral cavity cancers are amongst the most common cancers treated by the head and neck surgeon. It has been cited as the sixth most common cancer in the world with an estimate of close to 377,000 newly diagnosed patients in 2020 [1]. For curative intent, surgical ablation remains the modality of choice in most cases where patients are fit to undergo general anesthesia; alternative nonsurgical options such as chemotherapy and radiation are generally reserved for patients who are inoperable, unfit for anesthesia or in the palliative setting. Resection of these cancers demands a high level of clinical acumen from the surgeon to ensure resection of the tumor with a clear margin of greater than 5 mm in each three-dimensional direction. While clear surgical margins have been ubiquitously demonstrated to be one of the most consistent prognostic factors for local control and overall survival, excessive resection also has direct implications on a patient’s functional outcomes, and surgeons have to be judicious to achieve the best outcomes for their patients [2,3,4].

To date, despite the advances in preoperative imaging, the final resection margins are largely made following intraoperative decisions that often rely on a combination of visualization, clinical palpation and the surgeon’s experience. The infiltrative nature of these tumors within the depths of the oral cavity, compounded by the rigid skeletal framework surrounding it, makes access to, and therefore appreciation of, these margins difficult. It is thus not surprising that the occurrence of inadequate margins in oral cavity cancer surgery have has been reported to be as high as 85% [5,6].

Clearly the demands on the surgeon to achieve an ideal resection require significant training and experience. That being said, teaching this skill is difficult as the narrow surgical field often makes it technically challenging to demonstrate the decisions made in the intraoperative setting, and often surgical trainees have difficulty in clearly visualizing the surgical approaches during the resection. It is thus thought that, with the advances in technology and reality-based teaching scenarios, a surgical simulator may provide a better method for training surgeons on oral cavity ablation to allow for there to be a controlled environment for the intraoperative margin assessment. In addition to the surgical technique, a simulated tumor model would also allow surgical trainees to appreciate the different manners in which the tumor and margins are processed in the pathology lab. Thereby allowing them to gain a better understanding of the ways in which the histology reports are generated and the nuances of margin analysis from the pathology standpoint.

To date there is no well-defined surgical simulator that has been shown to be a reliable tool for training. The use of organic simulators such as ox tongues as a training model has been described for surgical training; however, the re-creation of an accurate tumor within these models is difficult [7]. An ideal surgical simulator would entail a configurable tumor that can accurately be placed within the model substance to allow for the precise production of a tumor model to achieve a standard conformance for surgical assessment following resection. This would allow for consistent analysis of a trainee’s resection margins for post simulation evaluation. Furthermore, this can be applied as a simulation model in the preoperative setting for complex cases involving oral cavity cancer ablation. The use of simulators has been shown to have significant benefits for trainees and has demonstrated to facilitate a significant skills transfer to the intraoperative setting [8,9]. Thus, undergoing a simulation prior to surgery could improve the trainees’ understanding and appreciation of the surgery, thereby maximizing the surgical experience and exposure.

A realistic simulator also has a potential role in the development of novel tools for use in oral cavity cancers. More recently, optical imaging technologies such as fluorescence imaging have been explored for surgical navigation, particularly in the head and neck. The development of these new technologies is often impeded by the lack of a realistic model to perform preclinical testing as a proof of concept. Conventional use of animal models such as bovine and porcine tongues are often unrealistic, while the use of fresh cadavers is costly and they are time consuming to obtain. The development of realistic models would allow for rapid testing of novel technologies in a consistent fashion for the purpose of intraoperative navigation in the treatment of oral cavity cancers. It was thus our aim to establish a structurally accurate model that would be able to simulate the surgical treatment of tongue cancers and compare this to current available organic models. This model would strive to act as both a model for surgeons in training as well as a vital research resource to evaluate the feasibility of navigation technologies in the preclinical setting.

## 2. Materials and Methods

Amongst the subsites of the oral cavity, tongue cancers are one of the most commonly encountered accounting for approximately 33.9–56.6% of all oral cavity cancers [10,11]. As such, a tongue cancer model was chosen as the first prototype in our aim to achieve a surgical simulation model for the oral cavity. We sought to compare this to a model that is commonly used for surgical simulation—a porcine tongue model. A waiver of IRB was obtained from the University Health Network (UHN) Research Ethics Board prior to the beginning of the study.

### 2.1. Construction of a Silicone Tongue Model

**Figure 1:**
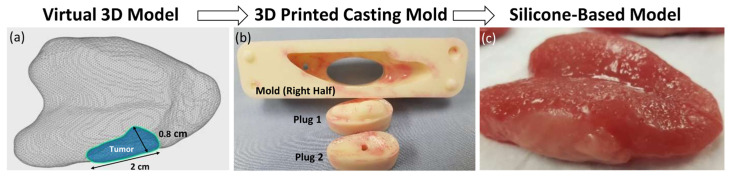
Tongue simulator fabrication process. (**a**) Virtual 3D model showing tumor (2 cm × 0.8 cm) embedded in tongue; (**b**) 3D printed mold and plugs for two-step casting (normal tongue then tumor); (**c**) silicone tumor model.

An overview of the multi-step process to fabricate the silicone simulator is shown in Figure 1. In order to establish a tongue model with customizable tumor volumes, head and neck computed tomography (CT) images of patients with normal tongues were identified and a three-dimensional (3D) segmentation was acquired from these images. For the purpose of this pilot study, a small tumor measuring 2.0 cm by 0.8 cm was selected as the tumor prototype and drawn within the normal tongue using 3D contouring software (ITK-SNAP 3.8, University of Pennsylvania, Philadelphia, PA, USA). The resultant normal tongue and tumor meshes were stretched in the anterior–posterior dimension by a factor of 1.5 using 3D mesh processing software [Meshmixer (Autodesk, San Francisco, CA, USA)]. This aimed to mimic the anterior pull of the tongue out of the oral cavity in the intraoperative setting (Figure 1a). The 3D segmentations were then used to fabricate an inverse mold using free 3D computer-aided design (CAD) software (OpenSCAD) and a 3D printer [3D Printer Dimension 1200es system (Stratasys, Eden Prairie, MN, USA)] with two plugs for casting normal tissue (Plug 1) and the tumor (Plug 2) (Figure 1b). Small 3D print striations were smoothed with a brushed epoxy coating [XCT-3D (Smooth-On, Easton, PA, USA)]. The mold was then filled in 2 stages with Smooth-On (Easton, PA, USA) silicone rubbers and additives of differing compositions to recreate the elastic and cutting properties of the tongue and tumor. First, the mold was filled with Ecoflex 00-30^®^ and Slacker^®^ to recreate muscle, followed by Dragon Skin 20^®^ for the tumor. Ecoflex 00-50^®^ was then applied to the exterior surface as mucosa. Each silicone rubber element had a cure time of 4 h. Mixtures of Silc-Pig^®^ silicone pigments (“Red”, “Blood” and “Flesh”) were added by eye (<0.1% by volume) for realistic presentation (Figure 1c). 

### 2.2. Construction of an Organic Tongue Model

**Figure 2:**
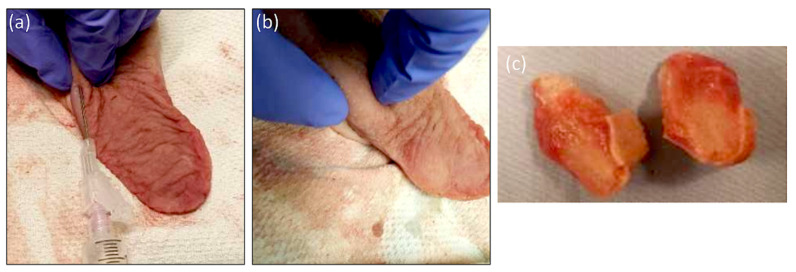
Porcine tongue model. (**a**) Injection of 2.5 cc of agar (2% solution) into porcine tongue; (**b**) palpation of tumor inclusion; (**c**) bifurcated specimen of tumor agar model in the pig tongue.

Pig tongues were obtained from a local butcher (Sumaq Wholesalers, Toronto, ON, Canada) to establish a biological comparison to the silicone model. In general, this is best performed within 7 days of obtaining the model. Following this, degradation of the soft tissue affects tissue quality during dissection. Using an 18-gauge needle, a 2% solution of agar was injected into the lateral aspect of the tongue in the submucosal layer to simulate a tumor (Figure 2a). Approximately 5–8 mL of agar was infiltrated to simulate a 2 cm tumor (Figure 2b,c). 

### 2.3. Evaluation of Training Model

To evaluate the model, participants with varying levels of experience were invited to perform tumor resections in the model. This included 4 senior residents, 4 fellows and 3 attending staff from the Department of Otolaryngology-Head and Neck Surgery at the University Health Network. Participants were each given a synthetic and a porcine tumor model and instructed to resect the tumor with clear margins. Participants were allowed to feel and examine the model prior to the surgical extirpation, and in the silicone model, MRI imaging [1.5T Magnetom Aera (Siemens Healthineers, Erlangen, Germany)] was also provided to simulate preoperative cross-sectional imaging. Imaging was not feasible for the porcine model due to the lack of delineation of the agar to the porcine tongue musculature. 

**Figure 3:**
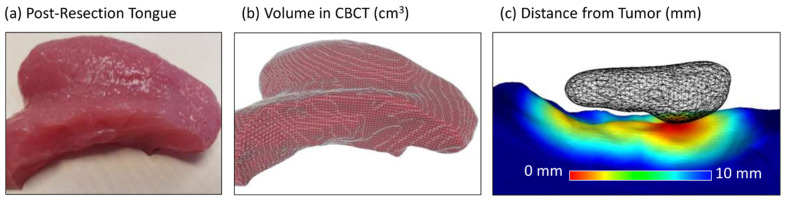
Post-resection analysis. (**a**) Silicone tongue model following tumor resection; (**b**) volumetric analysis of resection specimen on CT; (**c**) quantitative analysis of distance of resection surface from tumor inclusion.

Following resection, margins were then assessed by sectioning the removed specimen in conjunction with head and neck pathologists to evaluate the margin of clearance from the tumor. This was performed by gross evaluation of the resected specimen after sectioning. A portion of the specimen was sectioned in an enface manner while another portion of the specimen was evaluated by the “breadloaf” manner of sectioning. The distance between the edge of specimen to the edge of the tumor was then measured to determine the margins at each point. In addition to direct specimen evaluation, CT evaluation of margins could also be performed for the silicone model. Due to the uniformity of the silicone model, quantitative surgical margin analysis of the remanent tongue models (Figure 3a) was feasible using cone-beam CT (CBCT) imaging (Figure 3b). CBCT imaging was performed on a prototype flat-panel mobile C-arm developed for image-guided surgery [12]. Using ITK-SNAP software, mesh segmentations (STL format) were acquired by delineating the normal tongue and tumor on CT images of the original silicone model and the resection bed surface and normal tongue on CT images of participant’s post-resection models. These segmentations (pre- and post-operative) were then superimposed using an open-source software [MeshLab, Institute National Research Council, Sejong-si, Republic of Korea] implementation of iterative closest point (ICP) mesh registration to allow for a 3D evaluation of tumor margins including the deep margins [13]. The point-wise distance of the resection surface to the virtual tumor was then computed in MeshLab, and the minimum distance is reported below (Figure 3c).

To evaluate the models, we used a questionnaire focused on the face and content validity and the usefulness of the simulation as a training exercise for margin evaluation in oral cavity ablation. This was performed immediately upon completion of the session and all participants were asked to fill this out. This was performed anonymously, and participants were asked only to put in their level of training with no other identifiers. This questionnaire contained 3 questions each on face and content validity and 4 questions on the utility for application for medical training. All questions were scored from 1 to 5; 1 being poor to 5 being excellent. (Supplementary Materials) The mean scores of each of the domains were then compared between the 2 training models.

As part of a secondary analysis, we hypothesized that the surgical simulator would be able to distinguish between experienced and inexperienced surgeons. As such, we utilized data obtained from the quantitative analysis of the resection of the silicone model with a focus on the closest distance to tumor and compared this between residents (trainee surgeons) and those who have completed their residency training (fellows and attending surgeons). 

### 2.4. Statistical Analysis

Statistical analysis was performed using GraphPad Prism 7 (GraphPad Software, La Jolla, CA, USA). Mann–Whitney U test was used to compare the participants evaluation of both simulation models and a *p* value of <0.05 was deemed significant.

## 3. Results

### Validity of Models for Training

**Figure 4:**
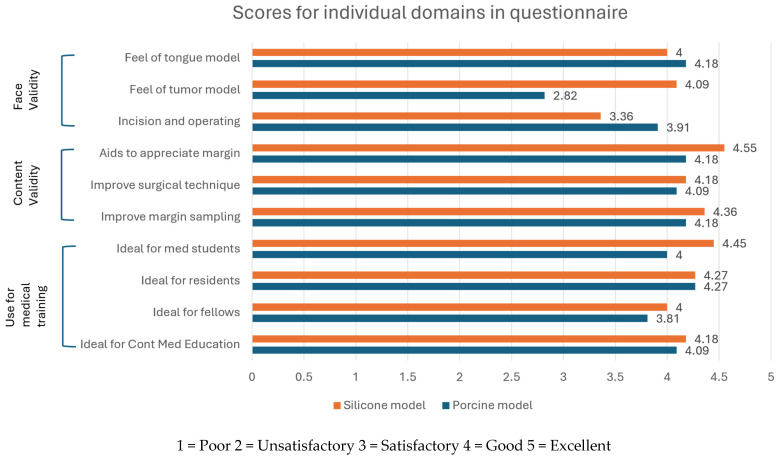
Individual domain scores from questionnaire grouped in different domains–face, content and utility in medical training.

The average face and content validity from the survey shows overall better scores (Table 1) for the silicone model when comparing it to the porcine model (mean face validity: 4 vs. 3.6, mean content validity: 4.4 vs. 4.1). Assessment of the individual domains from the questionnaire (Figure 4) allows for greater evaluation on each aspect of the model constructs. On evaluation of the handling of the tongue models, both the silicone and porcine models fared similarly (4.0 vs. 4.1). Tumor consistency, however, was far more realistic in the silicone construct compared to the porcine model (4.1 vs. 2.8, *p* = 0.0042). The assessment of realism in terms of the incision and operation demonstrated that there was better validity in the porcine model compared to that of the silicone model (3.9 vs. 3.6). When using a score of ≥4 as a cut-off for validity, the average face validity of the silicone model meant that it could be considered to be a valid model compared to the porcine model.

In the evaluation of the models’ use in training to determine surgical margins, the silicone model fared slightly better across all domains; this was, however, not statistically significant. Both models were valid in achieving the goals for the appreciation of margins during tongue cancer extirpation, this is supported by a ≥4 score across all domains in our survey. With the silicone model construct, we were further able to correlate surgical margins with CT imaging. Margin distance could then be segmented and presented for a comprehensive 3D analysis of margin clearance. This was useful as a pictorial guide for the appreciation of the 3D nature of the tumor and the related tumor margins. 

Using the data obtained from these margins, a comparison was performed between the groups to determine the margins based on training levels. This aimed to determine if the model would be able to differentiate clinicians by their experience, which would allude to the realism of the model. This was evaluated using the closest tumor margin obtained following an analysis of the resected specimens. This margin was then used to compare between residents and the attending surgeons and fellows (Table 2). In this analysis, the resident cohort obtained a median closest margin at 1.0 mm (range 0–3.5 mm) with the average closest margin reported at 1.4 mm. Within this group, one participant obtained a positive margin, while another obtained an extremely close margin at 0.1 mm. In contrast, the median closest margin in the attending/fellows group was measured to be 3.5 mm (range 1.4–4.5 mm) with the average closest margin reported at 3.0 mm. The closest margin reported within this group was 1.3 mm. The ability to distinguish the surgical outcomes based on clinical experience does support the realism of this model as a simulator. While this result was not statistically significant, this is likely due to the limited number of participants in this study.

## 4. Discussion

For a long time, surgical training has been likened to almost an apprenticeship whereby surgeons in training mirror the actions of the senior surgeon. As with any practical skillset, the need for continual practice and repetition allows for greater mastery over the procedure. While low-risk procedures are readily available for a surgical trainee to attain proficiency through repeated exposure, this is often less so for complex and oncological cases due to the inherent need to balance surgical training and patient safety/outcomes. 

As such, the majority of these ablations and the related intraoperative decisions are performed by the attending surgeon/fellows with assistance from surgical trainees. Often though, the surgical field is confined and a lot of the intraoperative decisions are determined by both visual and tactile feedback during the ablation which may not be fully appreciated by the assistants/surgical trainees. The lack of practice and the subsequent lack of confidence to perform these surgical tasks makes it difficult for surgical trainees to further expand their skills and likely leads to poorer outcomes when they begin their practice [14]. This is possibly further impacted by an overall decrease in training time due to reduced training hours and duty hour restrictions that are placed upon surgical residents [15,16,17]. Unfortunately, these factors do translate to patient outcomes and failure to obtain clear surgical margins does translate to a poorer survival outcome of up to 11–15% [18].

Medical training has come a long way, and a rigorous structured program is now the norm in most residencies. Most training programs today utilize cadaveric dissection as part of their curriculum in head and neck surgical training during residency. This exercise allows for a fundamental appreciation of surgical planes and an understanding of the anatomy that provides the building blocks for surgery. Cadaveric dissection is also an integral part of training as it reinforces the surgical approaches that one might use for surgery and imparts tissue handling skills. Employing cadavers for temporal bone dissection, endoscopic sinus surgery and neck dissections are ideal as often the general focus of these procedures is centered on an appreciation of anatomical landmarks. As a training resource for oncological ablation, it is, however, a poor model due to absence of tumor pathology. For this purpose, different skill sets are required that are not centered on anatomical recognition but rather on an ability to determine a safe margin from the tumor in the preoperative and the intraoperative setting. This often requires tactile evaluation of the submucosal extension of the tumor to ascertain an adequately deep margin. Furthermore, the majority of training centers adopt formalin-fixed cadavers for dissection workshops, which alters the normal anatomy and texture of tissues, especially that of the tongue. With these limitations, it is clear a better training tool is required in this field of training.

In recent years, advances in medical training have led to greater sophistication and realism in medical education. Amongst them, simulation has been shown to be an effective training tool in developing practical skills such as in the areas of resuscitation and laparoscopic procedures [19,20,21]. The ability to create a realistic model to facilitate surgical simulation has been shown to correlate with significant improvements in surgical training. Simulation training is not novel in otolaryngology and has been particularly prominent in the otology and rhinology subspecialities, where several studies have demonstrated the positive impacts of simulation models on mastoidectomy and skull base procedures in surgical training [22,23]. This is also reflected in the utilization of 3D printed models for pediatric airway management and laryngeal surgery [24,25,26,27]. The use of simulation models in head and neck oncology has, however, not been as readily adopted. This study therefore aimed to recreate a viable surgical model for oral cavity cancers to allow for surgical simulation and appreciation of the 3D structure of mucosal lesions to ensure adequate margins during ablation. Unique to head and neck oncology, tactile feedback is important in surgical management of oral cancers. As such, other simulation programs, i.e., virtual reality/augmented reality-based modalities, were felt to be inadequate in recreating the surgical environment. Therefore, the creation of a physical model was favored during the conception of this training simulation.

This pilot study demonstrates the utility of a simulation model for training surgeons in oral cavity ablation and margin analysis. Based on the feedback scores for content validity, all participants felt that this exercise was useful for gaining an appreciation of surgical margins. Between the two models, however, the silicone model fared better in demonstrating the nuances of intraoperative decisions to obtain clear margins. This was reinforced by improved scores in both domains regarding tumor margin assessment. This could have been attributed to a distinct advantage of the silicone model with its ability to review the participant’s surgical planes post resection in comparison to an “ideal” line of resection which enabled a comprehensive 3D analysis of the margins, especially of the deep margins, thereby allowing for feedback on the surgical ablation by each participant. In addition to this, participants had the benefit of the preoperative MRI imaging for the silicone model. As mentioned, this was not feasible in the porcine model due to a lack of definition and this is likely to contribute to the greater realism in surgical simulation seen between the two models. 

The mean face validity scores, which evaluate the construct of the models, invariably favored the silicone model, particularly with reference to the tumor construct. This was not surprising as the ability to control the interface between the “normal” tongue and the tumor allows for a subtle change in consistency that closely replicates the clinical feel during surgery. This further allows for an appreciation of a submucosal extension of tumor that is often difficult for less experienced surgeons to appreciate. Comparatively, this was not easy and at times not possible with the porcine model as the injection of the agar depended on multiple variables such as the thickness and consistency of the underlying muscle. With regard to the better scores for incision/operation and the feel of the tongue model, it is hypothesized that the porcine model fared better as the muscle fibers in the porcine model allowed for a more natural tensile reaction when performing the dissections compared to the silicone model. This is not unexpected and was a foreseeable limitation in the silicone model due to its inability to mimic the muscular fibers of an organic tongue. In the future, this may be reconstituted in the silicone model by the creation of small intervening bridges in the 3D mold to mimic the muscular fibers of the actual tongue. Certainly, another limitation of the model would be the lack of ability to appreciate the margins on the microscopic level; however, this was expected and is likely to be a limitation in all surgical models that would be difficult to overcome.

With regard to the target audience for this simulation exercise, the participants’ feedback indicated that this was more useful for those with less experience, i.e., medical students and residents. This was not unexpected, as the tumor model used in this exercise was fairly simple. However, it is hypothesized that a more complex tumor model may allow for greater benefits to the more advanced participants, i.e., senior residents and fellows. Furthermore, the use of a 3D printed mold could potentially create models of greater complexity, incorporating other components such as the mandible/maxilla that might be useful in preoperative assessment for both surgical ablation and reconstruction.

In the setting of medical education, cost remains a constant concern. Simulation training can incur a significant cost and this should certainly be evaluated before adoption. Clearly, the ability to construct this simulation model requires 3D printing facilities which would incur a significant cost. However, in the current setting, it is not uncommon for most teaching centers to have such 3D printing facilities. Furthermore, over the past decade, the cost of this technology has decreased significantly. Thus, excluding the costs of a 3D printer, we conducted a cost assessment between the fabrication of the silicone model and that of the porcine model to see which of the two models was more economically viable. Based on our overall cost analysis, the creation of a silicone model compared to a porcine model demonstrated greater cost savings when several models (15 models used for cost-analysis) were printed. Furthermore, unlike the porcine model, the silicone model did not require any period of thawing, did not require a cold room for storage, had a much longer shelf-life and was smaller and easier to store. 

A review of the literature demonstrates that a number of studies have looked at surgical simulations for head and neck oncology training. Clearly this is an area of interest for training centers, as is evident from the numerous publications and different approaches to developing these simulators. Recent articles have proposed revascularization of cadaveric models to further improve realism in surgical dissection with impressive face and content validity [28]. Other articles have reviewed the use of animal models, virtual reality and augmented reality approaches for head and neck and microsurgical training [29,30]. As discussed above, these studies are generally focused on technical capabilities for surgical access, tissue handling and recognition of surgical planes. These skill sets differ from oncological resection and intraoperative decision making, which is not feasible in cadaveric or animal simulations for the reasons mentioned above. In that setting, this is a novel simulator that can augment the current training tools. Another key advantage of this model when comparing it to cadaveric and animal models is its high fidelity and low cost, which are critical to the adoption of training materials for institutions around the world.

In addition to its use for training, the creation of a hyperrealistic model with properties that mimic the tongue/tumor interface will be extremely useful for testing different optical modalities that might potentially be used in surgical ablation of the oral cavity. Current methods include the use of animal models which have significant variability and lack accuracy. While the best option would be to go to the operating room at each instance, this is often cumbersome and inefficient. A realistic model allows for testing and optimization in the lab prior to its evaluation on-table which is efficient in both cost and time [31].

With the widespread availability of 3D printing and the ability to generate life-like phantom models, it is likely that this will impact surgical training in the future. This is particularly the case for complex, high-stake surgeries such as oral cancer ablations. This pilot study demonstrates the validity of using these tools to further improve training experiences to prepare surgeons better in appreciation of their surgical margins during oral cavity ablation. Further work will be required including the fabrication of the other subsites of the oral cavity and incorporation of the skeletal framework to establish a true intraoperative simulation to enhance the user’s experience. 

## 5. Conclusions

In conclusion, our study demonstrates a novel approach to surgical training for oral cavity ablation. It demonstrates a high-fidelity, low-cost training tool that can replicate surgery for oral cancers. Compared to conventional animal training models that have been used previously, the silicone model demonstrates improved overall face and content validity in surgical training. In particular, the ability to customize and design variable tumor types with precise submucosal extension allows trainee surgeons to develop clinical experience in intraoperative evaluation and improved decisions during oral cavity cancer ablation. Certainly, as this is a prototype, further development will be required to improve the construct; nonetheless, in its current form it allows for a different set of training skills to be practiced during the development of a head and neck surgical trainee.

## Figures and Tables

**Table 1:** Mean face and content validity scores for silicone and porcine models.

	Mean Face Validation	Mean Content Validation
Silicone model	4	4.4
Porcine model	3.6	4.1

**Table 2:** Individual participants’ closest margins registered on resection model and mean/median distances of closest margins differentiated between surgical experiences-residents vs. fellow/attending surgeons.

	Closest Margin Recorded		Closest Margin Recorded
Resident 1	0	Fellow/attending 1	3.2
Resident 2	1.8	Fellow/attending 2	1.3
Resident 3	3.5	Fellow/attending 3	3.9
Resident 4	0.1	Fellow/attending 4	4.5
		Fellow/attending 5	3.8
		Fellow/attending 6	1.4
	Median Distance of Closest Margin (mm)	Mean Distance of Closest Margin (mm)	
Residents	1	1.4	
Fellow/attendings	3.5	3	

## Data Availability

Study data is available upon reasonable request to the corresponding author.

## Supplementary Materials

The following supporting information can be downloaded at: https://www.mdpi.com/article/10.3390/jpm14050450/s1. Questionnaire evaluation of surgical simulation models.
