# Supplementary material for: Evaluation of a 3D Printed Silicone Oral Cavity Cancer Model for Surgical Simulations
Source: J Pers Med. 2024 Apr 25;14(5):450. doi: 10.3390/jpm14050450 (PMC11121819; doi:10.3390/jpm14050450)
Supplement: Supplementary file 1 [file jpm-14-00450-s001.zip › jpm-2962714-supplementary.pdf]

Questionnaire evaluation of surgical simulation models

UHN Princess Margaret Cancer Centre

Year of experience   Date

Model evaluated: Silicone model / Pig tongue model

FACE VALIDITY

Tongue model feels realistic	1	2	3	4	5
Tumor model feels realistic	1	2	3	4	5
Performing the Incision and operation feels realistic	1	2	3	4	5

1 = Poor 2 = Unsatisfactory 3 = Satisfactory 4 = Good 5 = Excellent

CONTENT VALIDITY

The simulation aids to appreciate margins in oral cancer surgery	1	2	3	4	5
The simulation improved my surgical techniques	1	2	3	4	5
The simulation improved my understanding of margin sampling	1	2	3	4	5

1 = Poor 2 = Unsatisfactory 3 = Satisfactory 4 = Good 5 = Excellent

UTILITY OF MODEL AS TRAINING TOOL

The model is ideal for medical student	1	2	3	4	5
The model is ideal for residents	1	2	3	4	5
The model is ideal for fellows	1	2	3	4	5
The model is ideal for ENT surgeons as part of continual education	1	2	3	4	5

1 = Poor 2 = Unsatisfactory 3 = Satisfactory 4 = Good 5 = Excellent
